# RETRACTED: Karnwal et al. Nanotechnology for Healthcare: Plant-Derived Nanoparticles in Disease Treatment and Regenerative Medicine. *Pharmaceuticals* 2024, *17*, 1711

**DOI:** 10.3390/ph19060839

**Published:** 2026-05-28

**Authors:** Arun Karnwal, Amar Yasser Jassim, Ameer Abbas Mohammed, Vikas Sharma, Abdel Rahman Mohammad Said Al-Tawaha, Iyyakkannu Sivanesan

**Affiliations:** 1 Department of Microbiology, Graphic Era (Deemed to be University), Dehradun 248009, India; arunkarnwal@gmail.com; 2 Department of Microbiology, School of Bioengineering and BioSciences, Lovely Professional University, Phagwara 144411, India; 3 Department of Marine Vertebrate, Marine Science Center, University of Basrah, Basrah 61001, Iraq; amar.yasser@uobasrah.edu.iq (A.Y.J.); amer.mohammed@uobasrah.edu.iq (A.A.M.); 4 Department of Molecular Biology and Genetic Engineering, School of Bioengineering and BioSciences, Lovely Professional University, Phagwara 144411, India; biotech_vikas@rediffmail.com; 5 Department of Biological Sciences, Al Hussein Bin Talal University Ma’an, Ma’an P.O. Box 20, Jordan; 6 Department of Environmental Health Science, Institute of Natural Science and Agriculture, Konkuk University, Seoul 05029, Republic of Korea

The journal retracts the review article titled “Nanotechnology for Healthcare: Plant-Derived Nanoparticles in Disease Treatment and Regenerative Medicine” [1], cited above.

Following publication, concerns were brought to the attention of the Editorial Office related to the authenticity and originality of the review article [1].

In accordance with the journal’s standard procedures, the Editorial Office and Editorial Board conducted an investigation that identified the presence of non-standard terminology and a substantial level of similarity in terms of text, phrasing, and structure to a prior publication [2], produced by a different authorship group, without appropriate reference to the original source. While the authors collaborated during the investigation, they were unable to satisfactorily clarify the above-mentioned concerns.

As a result, the Editorial Board has decided to retract this publication [1] as per MDPI’s retraction policy.

This retraction was approved by the Editor-in-Chief of the journal *Pharmaceuticals*.

The authors have been informed of this retraction and have indicated their disagreement with this decision.
